# Supplementary material for: Regulation of meiotic telomere dynamics through membrane fluidity promoted by AdipoR2-ELOVL2
Source: Nat Commun. 2024 Mar 14;15:2315. doi: 10.1038/s41467-024-46718-6 (PMC10940294; doi:10.1038/s41467-024-46718-6)
Supplement: Supplementary file 1 — Supplementary Information [file 41467_2024_46718_MOESM1_ESM.pdf]

Supplementary Information

Regulation of meiotic telomere dynamics through membrane fluidity promoted by AdipoR2-ELOVL2

Zhang. J, et al.

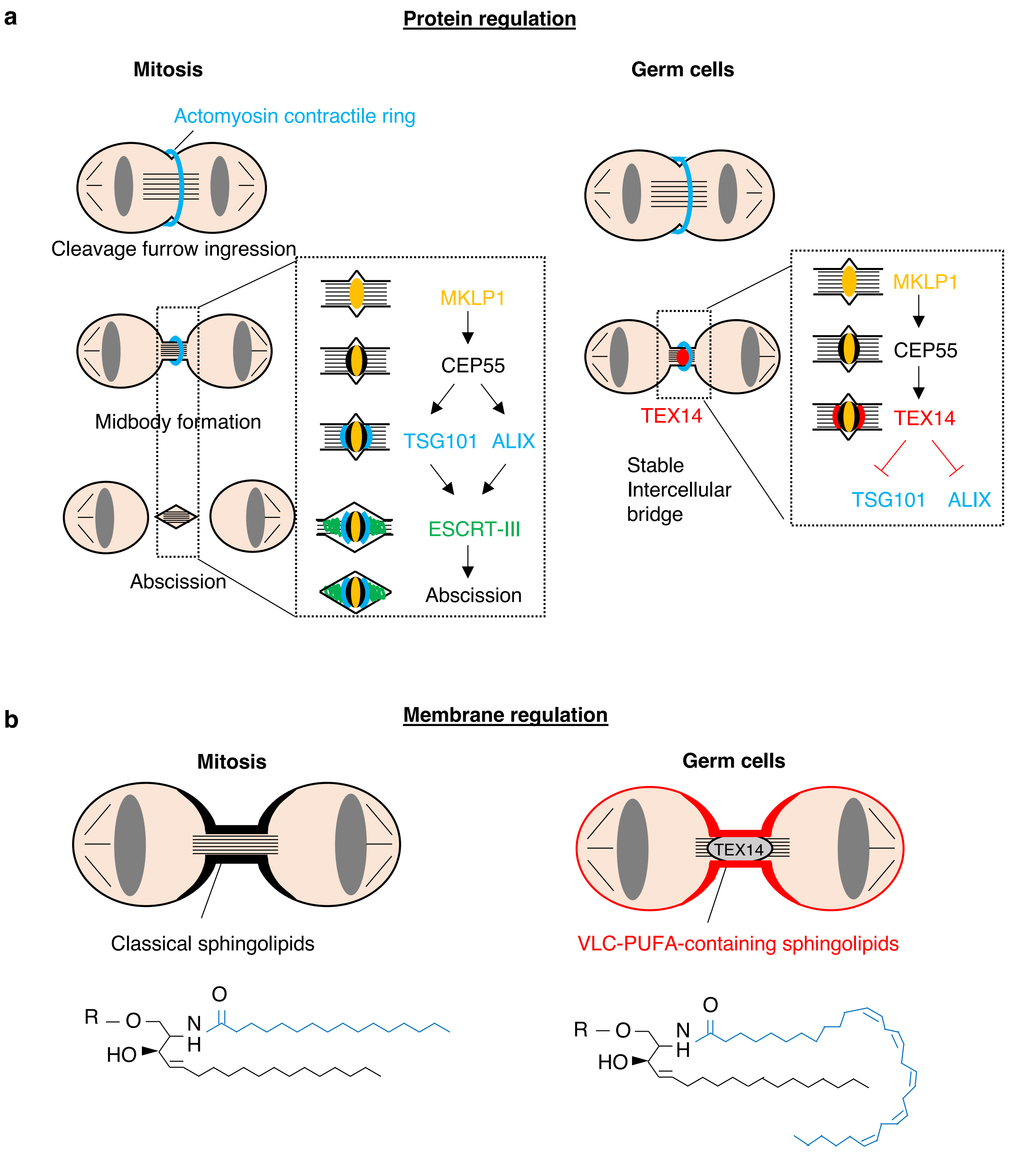

Supplementary Fig.1 : Schematic of cytokinesis regulations in mitosis and meiosis
(**a**) During mitosis, the midbody proteins MKLP1 and CEP55 recruit downstream effector proteins, such as TSG101, ALIX, and ESCRT-III, that are essential for cell abscission. However, in germ cells TEX14 binds directly to CEP55 and inhibits the recruitment of downstream effector proteins, thus stabilizing the temporal midbody structure and forming the intercellular bridge. (**b**) Midbody formation and the subsequent abscission in mitotic cells is regulated by the local rearrangement of the lipid membrane. In particular, classical sphingolipids as well as glycosphingolipids are enriched at midbodies prior to abscission. In germ cells, the formation of the intercellular bridge requires not only TEX14, but also specialized forms of sphingolipids that contain VLC-PUFAs.

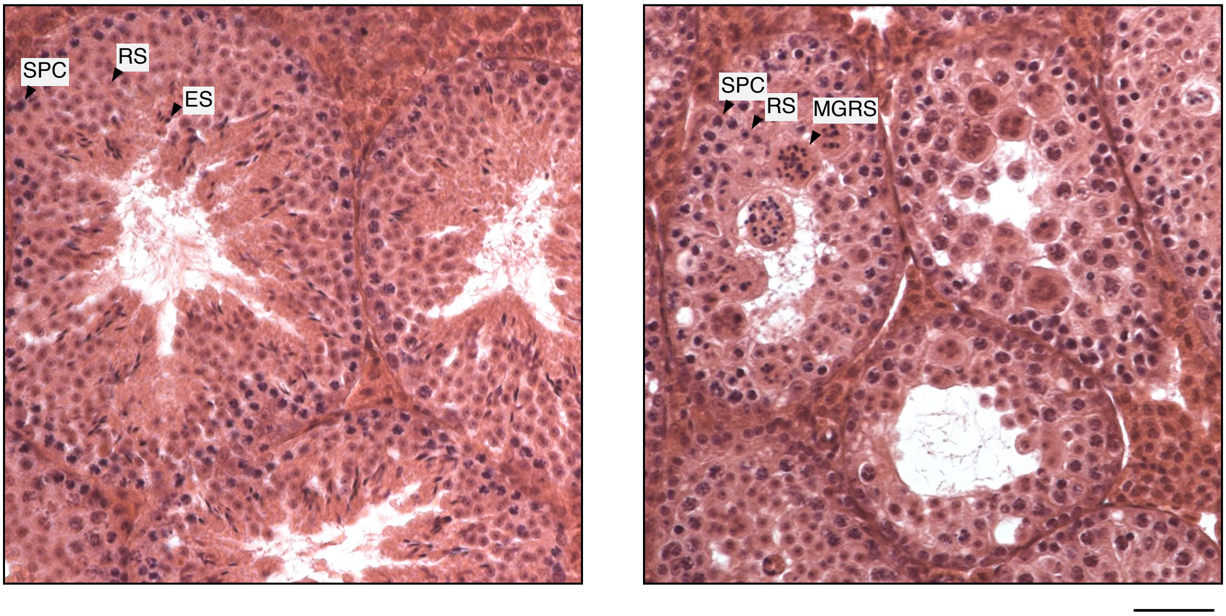

Supplementary Fig.2 : Spermatogenesis defects in *AdipoR2*$^{-/-}$ seminiferous tubules
Testis sections from 8-week-old WT and *AdipoR2*$^{-/-}$ males stained with hematoxylin and eosin. The arrowheads indicate a spermatocyte (SPC), round spermatid (RS), elongated spermatid (ES), and multinucleated giant round spermatid (MGRS). Scale bars: 50 µm. Experiments repeated in a total of 3 mice.

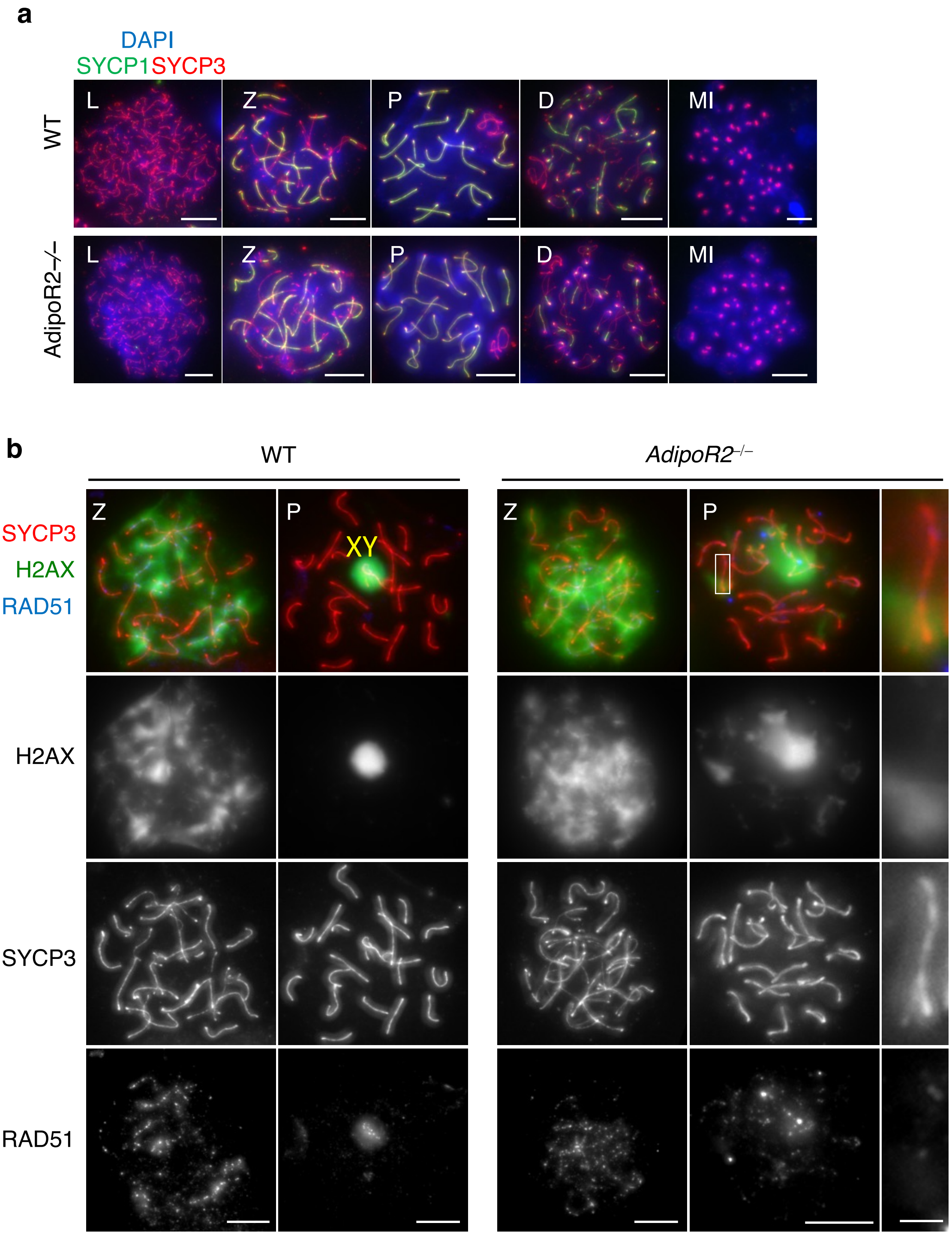

Supplementary Fig.3 : Synapsis defects in *AdipoR2*$^{-/-}$ pachytene spermatocytes

(**a**) Immunostaining of spermatocytes from WT and *AdipoR2*$^{-/-}$ male mouse testes. Spermatocytes were classified into the following substages: L, leptotene (no SYCP1); Z, zygotene (partially assembled SYCP1); P, pachytene (fully assembled SYCP1); D, diplotene (disassembled SYCP1); and MI, metaphase I (SYCP3 accumulations at centromeres). Scale bars: 5 µm. Experiments repeated in a total of 3 mice.

(**b**) Immunostaining of spermatocytes from WT and *AdipoR2*$^{-/-}$ male mouse testes. The localization of RAD51 foci was not observed along chromosome axes with Y-shaped branches in *AdipoR2*$^{-/-}$ pachytene spermatocytes. Scale bars: 5 µm. Experiments repeated in a total of 3 mice.

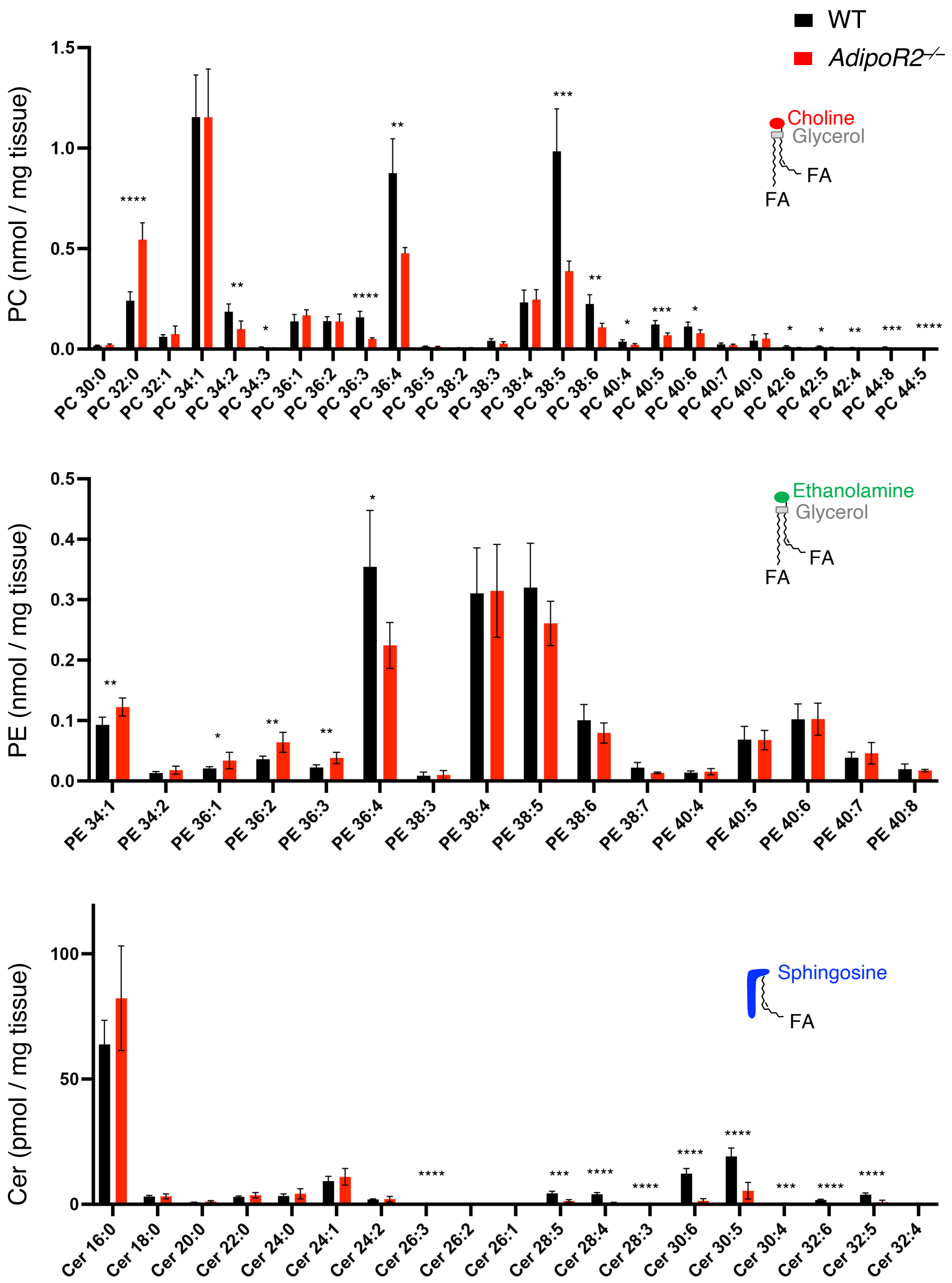

Supplementary Fig.4 : The depletion of VLC-PUFA in the in *AdipoR2*$^{-/-}$ testis

The amounts of each specified lipid species in the testes of PD28 male mice, both WT and *AdipoR2*$^{-/-}$, have been normalized to the tissue weight. n=7 and n =4 biological replicates were used for WT and *AdipoR2*$^{-/-}$, respectively. Mean values with SD are shown. Two-tailed t-tests. PC 32:0 $p < 0.0001$, PC 34:2 $p = 0.0060$, PC 34:3 $p = 0.018$, PC 36:3 $p < 0.0001$, PC 36:4 $p = 0.0014$, PC 38:5 $p = 0.00041$, PC 38:6 $p = 0.0012$, PC 40:4 $p = 0.016$, PC 40:5 $p = 0.00074$, PC 40:6 $p = 0.028$, PC 42:6 $p = 0.018$, PC 42:5 $p = 0.021$, PC 42:4 $p = 0.0023$, PC 44:8 $p = 0.00024$, PC 44:5 $p < 0.0001$, PE 34:1 $p = 0.0065$, PE 36:1 $p = 0.031$, PE 36:2 $p = 0.0020$, PE 36:3 $p = 0.0037$, PE 36:4 $p = 0.028$, Cer 26:3 $p < 0.0001$, Cer 28:5 $p = 0.00011$, Cer 28:4 $p < 0.0001$, Cer 28:3 $p < 0.0001$, Cer 30:6 $p < 0.0001$, Cer 30:5 $p < 0.0001$, Cer 30:4 $p = 0.00016$, Cer 32:6 $p < 0.0001$, Cer 32:5 $p < 0.0001$. Source data are provided as a Source Data file.

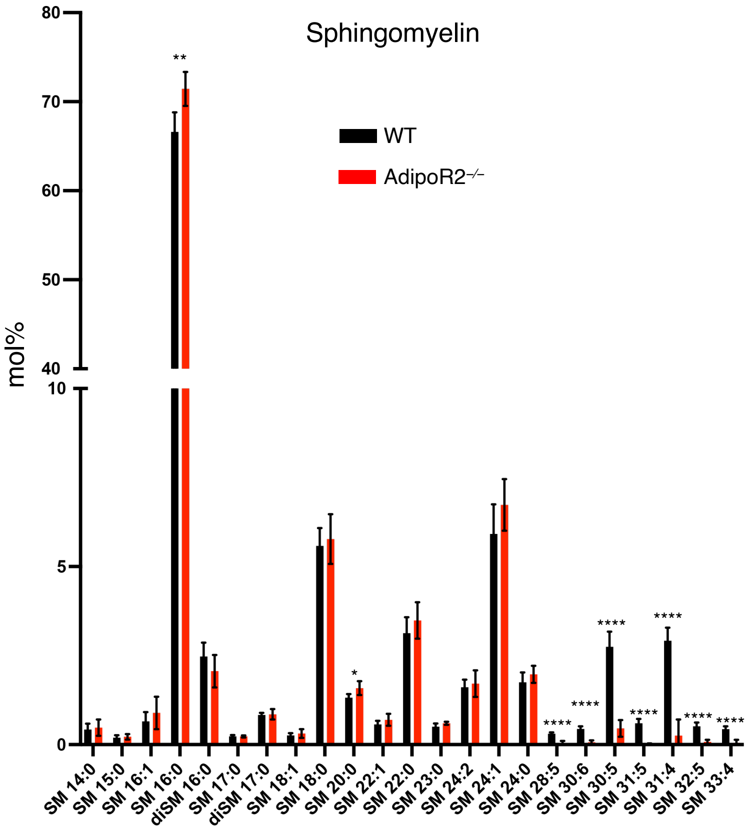

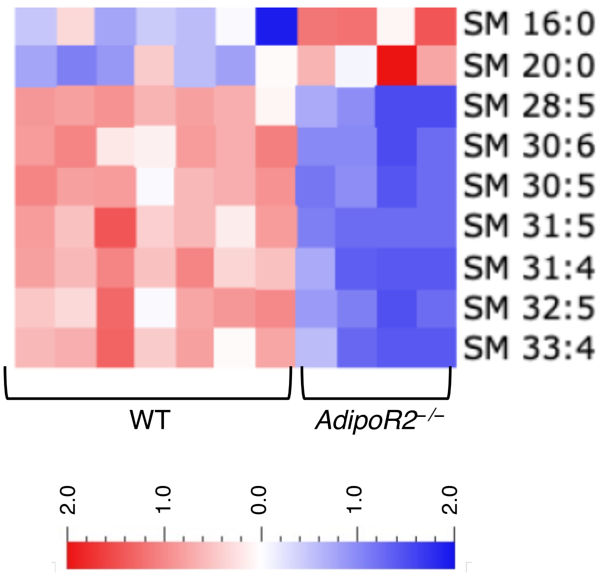

Supplementary Fig.5 : VLC-PUFA was depleted in sphingomyelins from *AdipoR2*$^{-/-}$ testes

The amounts of each indicated lipid species in PD28 testes from WT and *AdipoR2*$^{-/-}$ male mice shown as mol% (top). Data are shown as the mean with SD. Heat maps of the lipid species in PD28 testes from WT and *AdipoR2*$^{-/-}$ male mice (bottom). Only lipid species that showed significance with $p < 0.05$ are included in the heat map. Means for each lipid species were adjusted to 0, and the variance was adjusted to 1 in the heat maps. Two-tailed *t*-tests. SM 16:0 $p = 0.0052$, SM 20:0 $p = 0.013$, SM 28:5 $p < 0.0001$, SM 30:6 $p < 0.0001$, SM 30:5 $p < 0.0001$, SM 31:5 $p < 0.0001$, SM 31:4 $p < 0.0001$, SM 32:5 $p < 0.0001$, SM 33:4 $p < 0.0001$. n =7 and n =4 biological replicates were used for WT and *AdipoR2*$^{-/-}$, respectively. Source data are provided as a Source Data file.

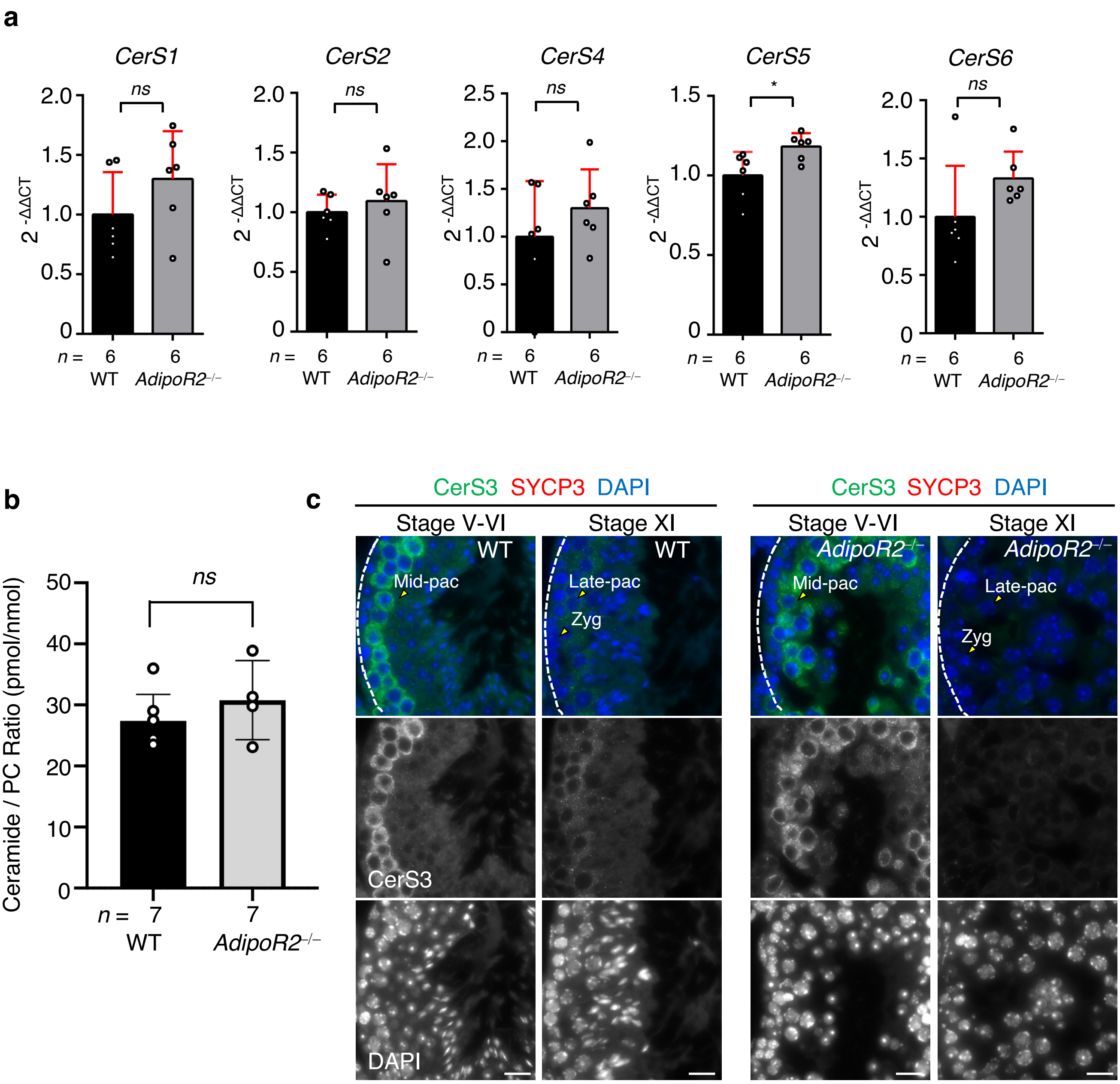

Supplementary Fig.6 : The synthesis of ceramides was not significantly affected in *AdipoR2*$^{-/-}$ testes

(**a**) Gene expression changes normalized to *GAPDH* in testes from WT and *AdipoR2*$^{-/-}$ male mice analyzed by qPCR. $n = 6$ testes pooled from three mice for each genotype. Two-tailed t-tests. ns: not significant. $^*p = 0.025$.

(**b**) The amount of total ceramide normalized to the total PC in WT and *AdipoR2*$^{-/-}$ male mouse testes at PD28. *n* shows the number of testis samples pooled from seven mice for each genotype. Data are shown as the mean with SD. Two-tailed *t*-tests. ns: not significant. n =7 and n =4 biological replicates were used for WT and *AdipoR2*$^{-/-}$, respectively.

(**c**) Immunostaining of testis sections from 8-week-old WT and *AdipoR2*$^{-/-}$ males. CerS3 signals were specifically detected in mid-pachytene spermatocytes (Mid-pac) in stage V–VI seminiferous tubules. CerS3 signals were hardly visible in zygotene (zyg) or late pachytene (Late-pac) spermatocytes in stage XI seminiferous tubules. Scale bars: 10 µm. Experiments repeated in a total of 3 mice. Source data are provided as a Source Data file.

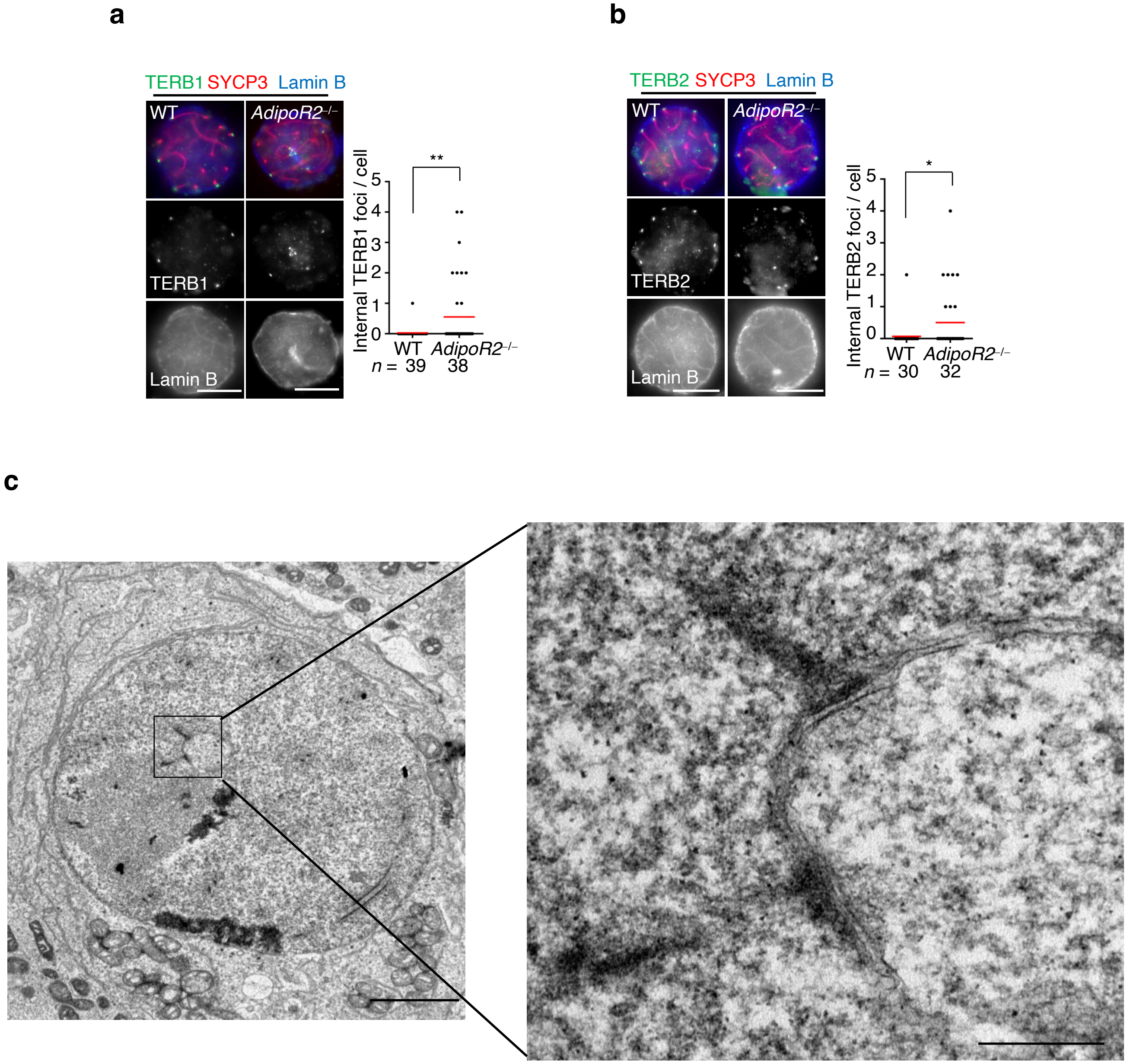

Supplementary Fig.7 : The telomere attachment defects in *AdipoR2*$^{-/-}$ testes
(**a**-**b**) Immunostaining of pachytene spermatocytes from WT and *AdipoR2*$^{-/-}$ male mice. Equatorial sections are shown. The graph shows the mean internal TERB1 (**a**) or TERB2 (**b**) foci number, and *n* shows the number of spermatocytes pooled from three mice for each genotype. Two-tailed *t*-tests. $*p = 0.033$, $**p = 0.0051$. Scale bars: 5 µm. (**c**) TEM images of spermatocyte from *AdipoR2*$^{-/-}$ male mice. Two telomeres attached to the invaginated NE are magnified. Scale bars: 2 µm (200 nm in the magnified panel). Experiments repeated in a total of 3 mice. Source data are provided as a Source Data file.

qPCR primers	Sequence (5'-3')
Gapdh-FOR	ACTCCCACTCTTCCACCTTC
Gapdh-REV	GGTCCAGGGTTTCTTACTCC
CerS3-FOR	ATCTCGAGCCCTTCTTCTCC
CerS3-REV	CTGGACGTTCTGCGTGAAT
Fads1-FOR	TGTGTGGGTGACACAGATGA
Fads1-REV	GTTGAAGGCTGATTGGTGAA
Fads2-FOR	CACCGACATTTCCAACAC
Fads2-REV	GGGCAGGTATTTCAGCTTCTT
Scd1-FOR	TCAACTTCACCACGTTCTTCA
Scd1-REV	CTCCCGTCTCCAGTTCTCTT
Elovl2-FOR	GACGCTGGTCATCCTGTTCT
Elovl2-REV	GCTTTGGGGAAACCATTCTT
Elovl4-FOR	TTCACTCTGTGGTGGATTGG
Elovl4-REV	AGCATGGTCAGGTATCGCTT
Elovl5-FOR	CTCTCGGGTGGCTGTTCTT
Elovl5-REV	AGAGGCCCCTTTCTTGTTGT
CerS1-FOR	TGACTGGTCAGATGCGTGA
CerS1-REV	TCAGTGGCTTCTCGGCTTT
CerS2-FOR	TCATCATCACTCGGCTGGT
CerS2-REV	AGCCAAAGAAGGCAGGGTA
CerS4-FOR	TGCGCATGCTCTACAGTTTC
CerS4-REV	CTCGAGCCATCCCATTCTT
CerS5-FOR	TCCATGCCATCTGGTCCTA
CerS5-REV	TGCTGCCAGAGAGGTTGTT
CerS6-FOR	GGGTTGAACTGCTTCTGGTC
CerS6-REV	TTTCTTCCCTGGAGGCTCT
**RT-PCR primers**	**Sequence (5'-3')**
Gapdh-RT-FOR	TTCACCACCATGGAGAAGGC
Gapdh-RT-REV	GGCATGGACTGTGTGGTCATGA
AdipoR1-RT-FOR	GGAGTGTTCCTGGGACTTGG
AdipoR1-RT-REV	TCTGATGAGACTGGAACCAGATG
AdipoR2-RT-FOR	GGAGATTTGGAGCCCAGCTT
AdipoR2-RT-REV	GGCCTTCCCACACCTTACAA

Supplementary Table1: The primers designed for this study
