# Supplementary material for: Regulation of meiotic telomere dynamics through membrane fluidity promoted by AdipoR2-ELOVL2
Source: Nat Commun. 2024 Mar 14;15:2315. doi: 10.1038/s41467-024-46718-6 (PMC10940294; doi:10.1038/s41467-024-46718-6)
Supplement: Supplementary file 3 — Reporting Summary [file 41467_2024_46718_MOESM3_ESM.pdf]

nature portfolio

Corresponding author(s): Hiroki Shibuya, Marc Pilon

Last updated by author(s): Feb 21, 2024

Reporting Summary

Nature Portfolio wishes to improve the reproducibility of the work that we publish. This form provides structure for consistency and transparency in reporting. For further information on Nature Portfolio policies, see our Editorial Policies and the Editorial Policy Checklist.

Statistics

For all statistical analyses, confirm that the following items are present in the figure legend, table legend, main text, or Methods section.

n/a	Confirmed	
☐	☒	The exact sample size (n) for each experimental group/condition, given as a discrete number and unit of measurement
☐	☒	A statement on whether measurements were taken from distinct samples or whether the same sample was measured repeatedly
☐	☒	The statistical test(s) used AND whether they are one- or two-sided<br>*Only common tests should be described solely by name; describe more complex techniques in the Methods section.*
☒	☐	A description of all covariates tested
☒	☐	A description of any assumptions or corrections, such as tests of normality and adjustment for multiple comparisons
☐	☒	A full description of the statistical parameters including central tendency (e.g. means) or other basic estimates (e.g. regression coefficient) AND variation (e.g. standard deviation) or associated estimates of uncertainty (e.g. confidence intervals)
☐	☒	For null hypothesis testing, the test statistic (e.g. F, t, r) with confidence intervals, effect sizes, degrees of freedom and P value noted<br>*Give P values as exact values whenever suitable.*
☒	☐	For Bayesian analysis, information on the choice of priors and Markov chain Monte Carlo settings
☒	☐	For hierarchical and complex designs, identification of the appropriate level for tests and full reporting of outcomes
☒	☐	Estimates of effect sizes (e.g. Cohen's d, Pearson's r), indicating how they were calculated

Our web collection on statistics for biologists contains articles on many of the points above.

Software and code

Policy information about availability of computer code

Data collection	Images were obtained on a microscope (Olympus IL-X71 Delta Vision; Applied Precision) equipped with 100× NA 1.40 and 60× NA 1.42 objectives, a camera (CoolSNAP HQ; Photometrics), and softWoRx 5.5.5 acquisition software (Delta Vision). For the measurement of membrane packing, images were acquired with an LSM880 confocal microscope (Zeiss) equipped with a live cell chamber (set at 33°C and 5% CO2) and ZEN software with a 40× water-immersion objective.
Data analysis	Images were analyzed by softWoRx 5.5.5 acquisition software (Delta Vision). All acquired images were processed with Photoshop (Adobe).

For manuscripts utilizing custom algorithms or software that are central to the research but not yet described in published literature, software must be made available to editors and reviewers. We strongly encourage code deposition in a community repository (e.g. GitHub). See the Nature Portfolio guidelines for submitting code & software for further information.

Data

Policy information about availability of data

All manuscripts must include a data availability statement. This statement should provide the following information, where applicable:

- Accession codes, unique identifiers, or web links for publicly available datasets
- A description of any restrictions on data availability
- For clinical datasets or third party data, please ensure that the statement adheres to our policy

The authors declare that the data supporting the findings of this study are available within the paper and its Supplementary Information. Source data are provided with this paper. All other data supporting the findings of this study are available from the corresponding author upon reasonable request.

Research involving human participants, their data, or biological material

Policy information about studies with human participants or human data. See also policy information about sex, gender (identity/presentation), and sexual orientation and race, ethnicity and racism.

Reporting on sex and gender	There is no human research participant in this study.
Reporting on race, ethnicity, or other socially relevant groupings	There is no human research participant in this study.
Population characteristics	There is no human research participant in this study.
Recruitment	There is no human research participant in this study.
Ethics oversight	There is no human research participant in this study.

Note that full information on the approval of the study protocol must also be provided in the manuscript.

Field-specific reporting

Please select the one below that is the best fit for your research. If you are not sure, read the appropriate sections before making your selection.

- [x] Life sciences
- [ ] Behavioural & social sciences
- [ ] Ecological, evolutionary & environmental sciences

For a reference copy of the document with all sections, see nature.com/documents/nr-reporting-summary-flat.pdf

Life sciences study design

All studies must disclose on these points even when the disclosure is negative.

Sample size	No statistical method was used to predetermine sample size. The sample sizes are described in the figures or the corresponding figure legends. Results were consistent across replicates.
Data exclusions	No data was excluded.
Replication	Each conclusion in the manuscript was based on results that were reproduced in at least two independent experiments and in at least two independent mice of each genotype.
Randomization	Mice were categorized based on their genotypes. The genotypes were determined by PCR.
Blinding	The investigators were not blinded to allocation during the experiments or to outcome assessment.<br>This is because the phenotypes were quite obvious that observer can be sure without blind test.<br>Further, the observer unbiasedly and carefully performed the quantification with enough sample number to make sure the conclusion.

Reporting for specific materials, systems and methods

We require information from authors about some types of materials, experimental systems and methods used in many studies. Here, indicate whether each material, system or method listed is relevant to your study. If you are not sure if a list item applies to your research, read the appropriate section before selecting a response.

Materials & experimental systems

n/a	Involved in the study
☐	☒ Antibodies
☐	☒ Eukaryotic cell lines
☒	☐ Palaeontology and archaeology
☐	☒ Animals and other organisms
☒	☐ Clinical data
☒	☐ Dual use research of concern
☒	☐ Plants

Methods

n/a	Involved in the study
☒	☐ ChIP-seq
☒	☐ Flow cytometry
☒	☐ MRI-based neuroimaging

Antibodies

Antibodies used

The following antibodies were used: rabbit antibodies against AdipoR2 (1:800 for WB, Marc Pilon lab), TEX14 (1:1000 for WB and 1:200 for IF, Abcam; Ab41733, GR109649-1), SYCP1 (1:5000 for IF, Abcam; Ab15090, GR3184119-1), γH2AX (1:3000 for IF, Abcam; Ab11174, GR294890-8), RAD51 (1:500 for IF, Thermo Fisher Scientific; PA5-27195, UI2840658J), ELOVL2 (1:1000 for WB, Abcam; Ab176327, GR139744-4), CerS3 (1:1000 for WB and 1:100 for IF, Roger Sandhoff lab), SUN1 (1:500 for IF, Abcam; Ab103021, 1014380-1), KASH5 (1:1000 for IF, Hiroki Shibuya lab), MAJIN (1:1000 for IF, Hiroki Shibuya lab), TERB1 (1:1000 for IF, Hiroki Shibuya lab), and TERB2 (1:1000 for IF, Hiroki Shibuya lab); rat antibody against RPA2 (1:200 for IF, Cell Signaling Technology; 2208S, 3); mouse antibodies against TRF1 (1:1000 for IF, Hiroki Shibuya lab), MLH1 (1:50 for IF, BD Biosciences; 51-1327GR, 4136717), and β-Actin (1:2000 for WB, Sigma; A2228-200UL, 067M4856V); goat antibody against Lamin B (1:100 for IF, Santa Cruz Biotechnology; sc-6216, F1715); and chicken antibody against SYCP3 (1:5000 for IF, Hiroki Shibuya lab).

Validation

Antibodies generated in Shibuya Lab, Pilon lab, and Sandhoff lab were validated by western blotting (WB) as well as immunofluorescence analysis (IF) using mouse testis samples.

The following commercial antibodies have been validated in the corresponding studies:

TEX14 (Abcam; Ab41733): Mouse, IF (PMID: 26045466)
SYCP1 (Abcam; Ab15090): Mouse, IF (PMID: 34075040)
γH2AX (Abcam; Ab11174): Mouse, IF (PMID: 30760716)
RAD51 (Thermo Fisher Scientific; PA5-27195): Mouse, IF and WB (PMID: 32345962)
ELOVL2 (Abcam; Ab176327): Human and mouse, WB (manufacturer's website: https://www.abcam.co.jp/products/primary-antibodies/elovl2-antibody-epr11880-ab176327.html)
SUN1 (Abcam; Ab103021): Mouse, IF (PMID: 31724724)
RPA2 (Cell Signaling Technology; 2208S, 3): Mouse, IF and WB (PMID: 32345962)
MLH1 (BD Biosciences; 51-1327GR): Mouse, IF, (PMID: 32345962)
β-Actin (Sigma; A2228-200UL): Mouse, WB, (PMID: 24818823)
Lamin B (Santa Cruz Biotechnology; sc-6216): Mouse, IF (PMID: 35081355)

Eukaryotic cell lines

Policy information about cell lines and Sex and Gender in Research

Cell line source(s)	Mouse cell line: C2C12 (Sigma, Cat#91031101-1VL)
Authentication	These cell lines are authenticated in the company (Sigma).
Mycoplasma contamination	Cell lines were not tested for the Mycoplasma contamination.
Commonly misidentified lines (See ICLAC register)	not applicable

Animals and other research organisms

Policy information about studies involving animals; ARRIVE guidelines recommended for reporting animal research, and Sex and Gender in Research

Laboratory animals

We used WT and genetically modified mice (AdipoR2 KO). All WT and knockout mice were congenic with the C57BL/6J background. We used adult (2 months old) male mice for most of the experiments, otherwise indicated in the figure legends.The mice are housed in IVC cages with a 12 hour dark and light cycle. The temperature is 20-22 °C and the relative humidity is between 45 and 60 %. The mice have bedding material in the form of wood shavings and wood litter as well as a house of paper and nesting pads as enrichment. Cage changing is done at least once a week. All animal experiments were approved by the Regional Ethics Committee of Gothenburg, governed by the Swedish Board of Agriculture (#1316/18).

Wild animals

No wild animal was used.

Reporting on sex	We studied the progression of male germ cell development. Thus, most of the data were obtained by using male mice. We used female mice only for the breeding and fertility assay.
Field-collected samples	Our study did not involve samples collected from the field.
Ethics oversight	All animal experiments were approved by the Regional Ethics Committee of Gothenburg, governed by the Swedish Board of Agriculture (#1316/18).

Note that full information on the approval of the study protocol must also be provided in the manuscript.

Plants

Seed stocks	There is no plant sample used in this study.
Novel plant genotypes	There is no plant sample used in this study.
Authentication	There is no plant sample used in this study.
