# Supplementary material for: Regulation of meiotic telomere dynamics through membrane fluidity promoted by AdipoR2-ELOVL2
Source: Nat Commun. 2024 Mar 14;15:2315. doi: 10.1038/s41467-024-46718-6 (PMC10940294; doi:10.1038/s41467-024-46718-6)
Supplement: Supplementary file 4 — Description of Additional Supplementary Files [file 41467_2024_46718_MOESM4_ESM.pdf]

Description of Additional Supplementary files

File name: Supplementary Movie 1.

Description: 3D reconstruction of WT round spermatids. Round spermatids from WT testis section were stained with SYCP3 (red), TEX14 (green), and DAPI (blue).

File name: Supplementary Movie 2.

Description: 3D reconstruction of AdipoR2−/− round spermatids. Round spermatids from AdipoR2−/− testis section were stained with SYCP3 (red), TEX14 (green), and DAPI (blue). (Example 1 of 2.)

File name: Supplementary Movie 3.

Description: 3D reconstruction of AdipoR2−/− round spermatids. Round spermatids from AdipoR2−/− testis section were stained with SYCP3 (red), TEX14 (green), and DAPI (blue). (Example 2 of 2.)
